# New frontiers and applications of attachment theory

**DOI:** 10.3389/fpsyg.2015.00273

**Published:** 2015-03-11

**Authors:** Silvia Salcuni

**Affiliations:** Dipartimento di Psicologia dello Sviluppo e della Socializzazione, Università degli Studi di PadovaPadova, Italy

**Keywords:** attachment, prevention, intervention, neuroscience, AAP, addiction, trauma, autism

Attachment is a deep and enduring emotional bond that connects one person to another across time and space (Bowlby, 1969; Ainsworth, 1973). Bowlby considered the importance of children's relationship with their mothers in terms of their social, emotional and cognitive development. Specifically, he emphasized the importance of the link between early infant separations from their mothers and related traumatic experiences and later maladjustment. His attachment theory provides a framework able to explain how the parent-child relationship emerges and influences subsequent development, becoming the key determinant of child's social emotional adaptation and cognitive growth (Belski and Fearon, 2002; Bernier and Meins, 2008).

During the last 20 years, attachment theory played an important role in the field of assessment, interventions and psychotherapy, representing the foundation of a wide range of perspectives and practices. Among others, some of such perspectives and practices stand out for their relevant consequences and effects, such as (1) the role of traumatic or neglectful bonds with early caregivers; (2) the importance of assessing patient's attachment pattern, attachment defenses and quality of affect regulation as to plan both individual therapies and dyadic/triadic interventions aimed to promote psychological development and wellbeing in children and adults; (3) the importance of giving the patient a close, intense, emotional and consistent therapeutic relationship (Wallin, 2007). Working within the framework of attachment theory has the ultimate goal of recreating in interventions or in psychotherapeutic treatment an attachment experience “that makes up, at least to some degree, for what the client missed the first time around” (Wylie and Turner, 2011, p.1). In the last years, integration between attachment and neuroscience underlined how “the art of a certain specialized form of relationship and attuned connection, isn't just helping people feel better, but deeply changing the physical function and structure of their brains as well” (Schore and Schore, 2008, 2010; Wylie and Turner, 2011, p. 17; Schore and Newton, 2012).

The present E-book is an interesting collection of original theoretical models, empirical papers in both clinical and non-clinical populations, and single case studies, which provide evidence for the need to move psychological comprehension toward the framework of the attachment theory. All papers share some common issues: first of all, the importance of assessing attachment to improve knowledge about patient's deep and inner relational functioning, emotional regulation and adjustment; second, the main aim is always to use these attachment notions within a multi-method assessment, able to strengthen incremental validity of results and interpretations; and, last but not least, the need to consider not just global attachment classifications (secure, dismissing, preoccupied, and disorganized), but also the implicit dimensions which lead to such general classification, such as coherence of mind, reflective function, evidence of secure base, capacity to act, defensive mechanisms: all these dimensions can offer more relevant information about the patient's inner world. Since each attachment pattern occurs in a unique way, a deeper knowledge about its functioning could provide clinicians with a personalized key of interpretation for each single patient, helping to establish a synchronized and responsive connection, in order to offer a second chance for the construction of a secure base (Wylie and Turner, 2011).

The E-book starts with an interesting paper written by Schore (2014), in which the author proposes an interpersonal neurobiological theoretical model of early socio-emotional development both for typical and a-typical (autistic) child development. The manuscript is an original work in line with Schore's contributions to affective interactive regulation and neuroscience, and suggests how recent literature on social and emotional functions of the early developing right brain may not only bridge the separate worlds of attachment and autism, but also facilitate more effective attachment and autism models of early intervention.

A series of original studies on mother-child interaction come next: these papers focus on the exploration of mother-child interaction effects in at-risk families (De Falco et al., 2014), in mothers who presented an unresolved trauma (Iyengar et al., 2014), and in drug addicted mothers who were hosted in a therapeutic community with their children (De Palo et al., 2014). Within a multi-method assessment framework, these papers provide insightful indications both of inclusion criteria for intervention programs and of treatment planning to support parenting skills in these specific mother-child dyads.

Other two papers present original research, which focuses on the assessment of attachment in people with eating dysregulation or disorder, using the Adult Attachment Projective Picture System (George and West, 2012). Delvecchio et al. (2014) paper describes a new conceptual model of attachment to understand the role of defensive exclusion in the development of early relationships. Such model is related to subsequent manifestations of symptoms of anorexia. Mazzeschi et al. (2014) manuscript demonstrated the role of paternal and maternal attachment status in the genesis and in the maintenance of childhood obesity. In line with the previous papers, these two contributions propose insightful implications for prevention and clinical intervention too.

A series of really interesting clinical case studies, mostly based on the use of the Adult Attachment Projective Picture System, together with other validated and powerful tools, follow this section and propose important attachment-based recommendations for intervention. First of all, George and Buchheim's (2014) manuscript demonstrates the advantages of using the AAP for the assessment of a traumatized patient in an inpatient setting. They present a case study to illustrate those AAP components that are particularly relevant to a psychodynamic conceptualization. A case reporting a high level of parental conflict is presented by Pazzagli et al. (2014), through the use of the AAP and the Circle of Security Parenting (COS-P); the authors offer specific clinical and contextual indications for the application of COS-P. In the paper devised by Salcuni et al. (2014), a clinical battery composed of AAP, Symptom Check List 90 Revised (SCL-90-R) and Shedler–Westen Assessment Procedure (SWAP–200) was administered in the pre and post assessment phases of a young adult panic disorder patient's psychotherapy; the authors outlined the reasons why they adopted an approach within a psycho-dynamic framework with specific attention to an “attachment theory stance” considered as the treatment of choice for this patient. An original interpretation of change in psychotherapy using the Psychodynamic Diagnostic Manual (PDM) was also included.

As a conclusion, an Opinion paper by Pace (2014) sheds light on the clinical and research perspectives of the Friend and Family Interview (FFI; Steele and Steele, 2005; Steele et al. unpublished manuscript), a novel method developed to assess the IWMs of young individuals, often used with adopted samples, pointing out to the necessity of considering not just the global attachment classifications (secure, dismissing, preoccupied and disorganized), but also other important FFI dimensions (coherence, reflective function, evidence of secure base, etc.) that could offer more relevant information about adoptees' inner world.

## Conflict of interest statement

The author declares that the research was conducted in the absence of any commercial or financial relationships that could be construed as a potential conflict of interest.
